# Special Issue “Molecular Studies on Plant and Plant In Vitro Systems Secondary Metabolism”

**DOI:** 10.3390/ijms27020645

**Published:** 2026-01-08

**Authors:** Andrey S. Marchev

**Affiliations:** Laboratory of Eukaryotic Cell Biology, Department of Biotechnology, The Stephan Angeloff Institute of Microbiology, Bulgarian Academy of Sciences, 139 Ruski Blvd., 4000 Plovdiv, Bulgaria; andrey.marchev@microbio.bas.bg

Plants have natural rich sources of various products and molecules of different chemical natures that are used to protect them from diseases, herbivore and insecticide attack, UV radiation, etc. [1,2]. These synthetized primary and, in particular, specialized metabolites (SMs) are of exceptional interest for humans due to their various health benefits, including anticancer, anti-inflammatory, antiaging, skin-altering, and anti-diabetic properties, among others [3,4]. These SMs are categorized into different chemical groups according to their biosynthetic pathways: terpenes (including volatile compounds, sterols, and carotenoids), polysaccharides, phenolic compounds, phytoalexins (sulfur-containing compounds), alkaloids (nitrogen-containing compounds), flavonoids, and hydrocarbons [5,6,7]. However, factors such as environmental (temperature, light, etc.) and geographical conditions, nutrient deficiency, and herbivore, fungi, or pest interactions contribute to the significantly large fluctuations in primary and SM accumulation in plants, leading to low and inconsistent yields. Therefore, to satisfy the needs of raw material supply for the food, cosmetic, or pharmaceutical industries, biotechnological approaches using plants’ in vitro systems offer an appealing alternative for the production of biologically active metabolites. Nevertheless, it is of crucial importance to understand the biosynthetic capacities and pathways, including the opportunities to enhance the SMs in plant and plant in vitro systems [8,9,10,11].

Based on this background, this Special Issue unites original articles and reviews that provide insight into plants’ SM accumulation under elicitor treatment and/or the influence of light; the phytochemical composition, antimicrobial, and antioxidant effects of Himalayan lichen; the biosynthetic pathways of plant-based biofungicides; as well as exploring the untapped potential of plant in vitro cultures (in particular hairy roots) for the biosynthesis of high-value SMs and bringing new insights in the biotechnology toolkit utilized to enhance SM production. In total, three articles and two reviews have been accepted for publication in this Special Issue.

The original work of Wiczkowski et al. (Contribution 1) describes the accumulation and composition of anthocyanins in leaves of *Kalanchoë blossfeldiana* Poelln. detached and kept for five days under natural light conditions. Using the micro-HPLC/MS/MS-TOF method, the presence of seven cyanidin, six delphinidin, and two petunidin derivatives were confirmed.

The study shows that the leaves of *K. blossfeldiana* contain a 15-component set of anthocyanins consisting of monoglycosides, diglycosides, triglycosides, their acyl derivatives, and four unknown derivatives of petunidin, cyanidin, and delphinidin. The positions in which the leaves were kept after being detached from the plant caused different responses of distinct types of anthocyanins. Among the anthocyanins present in *K. blossfeldiana*, delphinidin-rhamnoside-glucoside, cyanidin-rhamnoside-glucoside, and cyanidin-glucoside were responsive to the stress caused by detaching the leaves and keeping them for five days with the abaxial side exposed to light. It is very likely that these anthocyanins accumulate under the epidermis of the abaxial side to protect it from excessive light, and their presence causes the visible red coloration of the leaves. The leaf positioning arrangement did not affect the contents of mono-glycosides, tri-glycosides, and acyl derivatives of cyanidin and delphinidin. The effect from leaf positioning was ambiguous for the unknown derivatives of petunidin, cyanidin, and delphinidin. The application of methyl jasmonate (JA-Me) reduced the contents of delphinidin rhamnoside-glucoside, cyanidin-rhamnoside-glucoside, and cyanidin-glucoside-glucoside, but did not affect the contents of mono-glycosides and tri-glycosides. This effect was probably due to the presence of the aforementioned di-glycosides in the subepidermal cells of *K. blossfeldiana* leaves. These were directly exposed to JA-Me, leading to the inhibition of their biosynthesis and/or accumulation. The lack of a significant effect of JA-Me on the mono- and tri-glycosides of cyanidin and delphinidin may in turn indicate the presence of these anthocyanins in the mesophyll. Therefore, the authors concluded that the metabolic response in detached *K. blossfeldiana* leaves, which involved changes in anthocyanin composition and accumulation sites, was likely a combined effect of light stress and wounding.

The study by Cenobio-Galindo et al. (Contribution 2) provides a compilation of biosynthesis techniques, the mechanisms of action that SMs use against phytopathogens, extraction techniques and the formulation of biofungicides, the biological activity of plant extracts on phytopathogenic fungi, the regulations, advantages, disadvantages, and an overview of the current use of biofungicides in agriculture. These includes the biosynthesis of alkaloids, terpenes, and phenolic compounds. The mechanisms of action of plant-based biofungicides against phytopathogenic fungi are summarized in several categories, such as the inhibition of protein synthesis, DNA synthesis, electron transport, the destruction of mitochondria, and the destruction of the cell wall, membrane, and hyphae. The utilization of conventional and non-conventional ecological and selective methods for extraction have been described. By presenting several biofungicides in the control of phytopathogenic fungi, it is very clearly underlined that, due to the impact that synthetic fungicides have on the environment and health, the use of these products will need to be strictly regulated by governments in the near future, which may lead to an increase in demand for plant-based products. This makes it clear that plant extracts are effective, biodegradable, and not as harmful to the environment as the synthetic chemicals that are often used. The option of replacing chemicals with plant-based formulations fits appropriately with future-oriented food and agricultural policies. Therefore, the production of more biofungicides must become common practice, and the approval policy for their commercialization must also be regulated.

In the study by Manojlović et al. (Contribution 3), the phytochemical composition and antimicrobial, antibiofilm, and antioxidant effects of a newly described Himalayan lichen *Placidium deosaiense* Usman and Khalid grown in Pakistan were investigated. Five secondary metabolites—olivetol, olivetolic acid, haematommic acid, fallacinol, and parietin— were identified as major compounds in *Placidium deosaiense* Usman and Khalid using the HPLC-DAD method. This study also showed that the tested lichen has important quantities that are often associated with phenolic compounds. Parietin was isolated from the acetone extract. It was of particular importance to show the antimicrobial, antibiofilm, and antioxidant activities of this specific and new species, given that it grows under specific conditions at high altitudes. The isolated compound parietin demonstrated the strongest DPPH free-radical-scavenging activity (IC_50_ = 51.616 µg/mL) and the highest reducing capacity compared to the tested extracts of the lichen. Although the extracts showed the best antibacterial activity (especially against *Proteus mirabilis* ATCC12453), parietin demonstrated superior antibiofilm activity (especially against *Staphylococcus aureus* ATCC25923).

The study by Mvingu et al. (Contribution 4) constitutes a notable contribution to the comprehension of the phytochemical composition and pharmacological potential of three Congolese *Croton* species: *C. mubango*, *C. haumanianus*, and *C. sylvaticus*. Using classical phytochemical screening in combination with dereplication techniques, a large number of secondary metabolites have been successfully identified, with terpenoids as the major class. Specifically, diterpenoids, sesquiterpenoids, monoterpenoids, triterpenoids, and steroids, together with other minor secondary metabolites such as saponins, amino acid, and fatty acids, were identified. The findings indicate a unique chemical profile for *C. sylvaticus*, revealing previously unreported compounds in the species, such as acetyl aleuritolic acid, caryophyllene oxide, and phytol using nuclear magnetic resonance (NMR) spectroscopy. Several SMs were identified in the dichloromethane extract of trunk bark, including monoterpenoids, sesquiterpenoids, diterpenoids, triterpenoids, along with other minor metabolites such as steroids, saponins, and fatty acids. Further purification of this extract led to the isolation for the first time of three major secondary metabolites, acetyl aleuritolic acid, caryophyllene oxide, and phytol. The bioactivities of anticancer terpenoids from *C. mubango* and *C. haumanianus* were evaluated using molecular docking techniques. Some of the tested compounds were identified as having strong-to-moderate predicted binding affinities to both protein targets (the human androgen receptor (HAR, PDB ID: 1E3G) and hypoxia-inducible factor 1-alpha (HIF-1α, PDB ID: 3KCX)), along with favorable drug-like properties according to ADMET analysis. A structure–activity relationship (SAR) analysis revealed that molecular weight, hydrophobicity (log *p* values), and the distribution of hydrogen bond donors and acceptors play key roles in binding affinity. Therefore, not only the investigated compounds but also the approach itself could contribute to novel bioactive compounds and sustain drug discovery initiatives.

The contribution by Mirmazloum et al. (Contribution 5) explores the potential of hairy roots (HRs) for SMs biosynthesis, focusing on biotechnology tools to enhance their production. These plant in vitro systems are genetically stable and offer a biosynthetically consistent platform with a high growth and productivity rate. Strategies like nutrient medium optimization, precursor feeding, elicitation, and metabolic engineering (e.g., the editing of non-coding regulatory DNA/RNA sequences, the overexpression of biosynthetic genes and TFs, or the suppression of catabolic or competing pathway genes) have been employed to boost the biosynthesis of these low-volume, high-value SMs. Recent advancements in genomic engineering, particularly CRISPR/Cas9 technology, open new possibilities for modifying metabolic pathways and creating custom, high-yielding HR lines capable of producing functional molecules for application in the food, cosmetic, and pharmaceutical industries. The combination of CRISPR/Cas technology and non-coding regulatory DNA has significant potential to generate novel alleles that affect the various agronomic traits of crops or medicinal plants for the precise regulation of the biosynthesis of SMs. The major bottleneck that hampers the industrial production of SMs based on HR cultivation is the poor understanding of the metabolic pathways of SMs and their regulation, which further reflects on their competitive economic value. Therefore, future focus should be on the integration of different approaches, including omics (genomics, transcriptomics, proteomics, and metabolomics) technologies, to understand metabolite-synthesis pathways through the identification of missing/unknown metabolite pathway enzymes or to identify the enzyme variants with higher activities that are expected to obtain higher SM productivity.

The commercial exploitation of SMs requires scaling up the HRs in bioreactors. Considering the unique morphological peculiarities and sensitivity of HRs, suitable bioreactor designs must provide optimized oxygen transfer, adequate growth conditions, appropriate agitation and aeration, a homogeneous culture environment, and minimized shear stress. Various liquid-phase, gas-phase, and hybrid bioreactors with numerous modifications have been designed to meet these challenges. Additionally, the initial investment and overall operating costs during the cultivation process are correlated with the appropriate design of the bioreactor and the optimized process parameters. The use of disposable bioreactors is the preferred choice for reducing the production costs, while the application of mathematical models and real-time monitoring can be harnessed to optimize the cell-cultivation process and enhance the productivity in plant cell bioreactors at a commercial scale.
